# Current Status of Next-Generation Sequencing Approaches for Candidate Gene Discovery in Familial Parkinson´s Disease

**DOI:** 10.3389/fgene.2022.781816

**Published:** 2022-03-01

**Authors:** Nikita Simone Pillay, Owen A. Ross, Alan Christoffels, Soraya Bardien

**Affiliations:** ^1^ South African National Bioinformatics Institute (SANBI), South African Medical Research Council Bioinformatics Unit, University of the Western Cape, Bellville, South Africa; ^2^ Department of Neuroscience, Mayo Clinic, Jacksonville, FL, United States; ^3^ Department of Clinical Genomics, Mayo Clinic, Jacksonville, FL, United States; ^4^ Africa Centres for Disease Control and Prevention, African Union Headquarters, Addis Ababa, Ethiopia; ^5^ Division of Molecular Biology and Human Genetics, Department of Biomedical Sciences, Faculty of Medicine and Health Sciences, Stellenbosch University, Cape Town, South Africa; ^6^ South African Medical Research Council/Stellenbosch University Genomics of Brain Disorders Research Unit, Cape Town, South Africa

**Keywords:** Parkinson’s disease, next-generation sequencing, whole-exome sequencing, familial PD, african ancestry, bioinformatic pipelines, third-generation sequencing, diverse populations

## Abstract

Parkinson’s disease is a neurodegenerative disorder with a heterogeneous genetic etiology. The advent of next-generation sequencing (NGS) technologies has aided novel gene discovery in several complex diseases, including PD. This Perspective article aimed to explore the use of NGS approaches to identify novel loci in familial PD, and to consider their current relevance. A total of 17 studies, spanning various populations (including Asian, Middle Eastern and European ancestry), were identified. All the studies used whole-exome sequencing (WES), with only one study incorporating both WES and whole-genome sequencing. It is worth noting how additional genetic analyses (including linkage analysis, haplotyping and homozygosity mapping) were incorporated to enhance the efficacy of some studies. Also, the use of consanguineous families and the specific search for *de novo* mutations appeared to facilitate the finding of causal mutations. Across the studies, similarities and differences in downstream analysis methods and the types of bioinformatic tools used, were observed. Although these studies serve as a practical guide for novel gene discovery in familial PD, these approaches have not significantly resolved the “missing heritability” of PD. We speculate that what is needed is the use of third-generation sequencing technologies to identify complex genomic rearrangements and new sequence variation, missed with existing methods. Additionally, the study of ancestrally diverse populations (in particular those of Black African ancestry), with the concomitant optimization and tailoring of sequencing and analytic workflows to these populations, are critical. Only then, will this pave the way for exciting new discoveries in the field.

## Introduction

Over the past almost 2 decades, next-generation sequencing (NGS) approaches, with their high-throughput and rapid output, have accelerated novel gene discovery for several human diseases. In this Perspective article, we summarize, analyze and highlight the studies that identified new loci for Parkinson’s disease (PD) using NGS strategies.

PD is a neurodegenerative disorder, typically presenting with bradykinesia, rigidity, resting tremor, postural instability, and various non-motor symptoms (Kalinderi et al., 2016). Approximately 90% of PD cases are considered sporadic; attributed to synergistic interactions between genetic, metabolic and environmental factors (Ball et al., 2019). The remaining 5–10% of cases are accounted for by familial PD, usually displaying a Mendelian mode of inheritance (Lesage and Brice, 2012; Hernandez et al., 2016). Positional cloning approaches have been used successfully to identify disease genes within large multi-incident PD kindreds (Hildebrandt and Omran, 1999). Linked regions of the genome that co-segregated with disease were then Sanger sequenced to identify the causal variant. PD genes identified using this approach have demonstrated autosomal dominant (AD-PD) (*SNCA, LRRK2*), autosomal recessive (AR-PD) (*PRKN*, *PINK1, DJ1*) and X-linked (*RAB39B*) inheritance patterns (Bras and Singleton., 2011; Gasser, 2013; Bandres-Ciga et al., 2020).

Later, development of high-throughput genotyping techniques allowed for the rapid screening of single-nucleotide variants (SNVs) - that occur with moderate to high allele frequencies - in large case/control cohorts (Shulskaya et al., 2018). This resulted in the rise of genome-wide association studies (GWAS), and adoption of the common-disease-common-variant hypothesis, which has been responsible for the discovery of many PD-susceptibility loci (Hemminki et al., 2008; Nalls et al., 2019). Yet, it has also been postulated that the gaping ‘missing heritability’ in complex disorders such as PD, may be attributed to larger penetrant effects of less common variants i.e., the rare-variant-common-disease hypothesis (Gasser et al., 2011; El-Fishawy, 2013; Germer et al., 2019).

### Next-Generation Sequencing in PD

NGS, in the form of whole-exome sequencing (WES), captures only the coding region; while whole-genome sequencing (WGS) sequences the entire genome including all non-coding regions (Fernandez-Marmiesse et al., 2017). When considering NGS for the study of genetic disorders, WES presents as the more suitable choice as most pathogenic mutations (80–85%), found to date, are exonic (Ku et al., 2016). WES is also cheaper, and less computationally intensive than WGS (Bonnefond et al., 2010; Chakravorty and Hegde, 2017). However, WES can result in skewed coverage due to hybridization biases and incomplete target enrichment, making detection of copy number variation (CNV) challenging (Belkadi et al., 2015). Since CNVs encompassing complete exons (in *PRKN*, *PINK1* and *DJ-1*) or spanning multiple gene copies (*SNCA*) are a significant cause of PD, this is a notable limitation of WES in PD studies. Together, these factors indicate that WGS may be more effective for identification of novel or rare genetic variants, particularly in complex diseases like PD.

### Novel Gene Discovery in PD-Affected Families Using NGS

For our search, a comprehensive search string on NCBI’s PubMed Central database “((((((parkinson’s disease) AND NGS) AND familial) AND novel) AND candidate) AND gene)” was done on 13 May 2021. Abstracts were read to identify studies that specifically used NGS (either WES or WGS) approaches to identify potential novel genes in familial PD or parkinsonism. We did not exclude studies with a lack of evidence of pathogenicity, and this resulted in a total of 17 relevant studies. These studies and their approaches are summarized in Table 1 and are discussed in chronological order below.

**TABLE 1:** List of published studies that identified novel Parkinson’s disease loci using next-generation sequencing approaches.


Reference	Gene	Population	Pre-NGS screening approach used	Study Participants Screened (Sequencing platform used)	QC and Read Alignment Tools	Variant Calling Tools	Variant Annotation and *In Silico* Pathogenicity Prediction Tools	Variant Inclusion/Exclusion Criteria	Mutations Identified/(Chromosome)
Vilariño-Güell *et al.* (2011)	***VPS35* (vacuolar protein sorting 35 ortholog)**	Swiss family (Family A)	None	WES on a PD-affected pair of 1st degree cousins	SOAPaligner (read alignment to the human References genome - Hg18, build 36.1)	SOAPsnp (SNP calling)	Database of Genomic Variants v6 (determination of structural variants against CNVs)	Variants were excluded if	Homozygous c.1858G > A
								- on the X chromosome	- p.Asp620Asn (16q11.2)
								- homozygous (autosomal-dominant inheritance of disease was assumed)	
								- non-coding	
								- synonymous	
								- variants present in dbSNP v.130	
								Variants were subsequently genotyped in a multi-ethnic case-control series (4,326 patients and 3,309 controls)	
								Confirmation *via* Sanger sequencing	
Zimprich *et al.* (2011)	***VPS35* (vacuolar protein sorting 35 ortholog)**	Austrian family	Haplotyping and linkage analysis (Merlin software)	WES on two PD-affected second cousins (Genome Analyzer IIx system (Illumina)	Burrows-Wheeler Aligner (BWA version 0.5.8) (read alignment to human References genome - Hg19)	SAMtools (v 0.1.7)—(SNVs and InDel calling)	PolyPhen2, SNAP and SIFT—(pathogenicity prediction)	Variants were excluded if	Heterozygous c.1858G > A
								- present in the 72 control exomes of non-PD patients	- p.Asp620Asn (16q11.2)
								- present in dbSNP131 and 1000-Genomes Project	
								- had an average heterozygosity of more than 0.02	
								Variants were included if	
								- heterozygous	
								- non-synonymous	
Edvardson *et al.* (2012)	***DNAJC6* (DnaJ Heat Shock Protein Family (Hsp40) Member C6)**	Palestinian family (two patients and their unaffected brother)	Homozygosity mapping and SNP genotyping in a consanguineous family (SNP genotyping using Affymetrix GeneChip Human Mapping 250 K Nsp Array	WES on a single index patient (GAIIx, Illumina)	Burrows-Wheeler Aligner (BWA) (sequence reads were aligned to human References genome - hg18 (GRCh36))	Genome Analysis Toolkit (GATK) (variant calling)	ANNOVAR (variant annotation)	Variants were excluded if	Homozygous c.801–2A > G (1p31.3)
					Picard (marking of PCR duplicates)		SeattleSeq Annotation (GERP score)	- present in dbSNP132, 1000-Genomes Project and in-house databases	
							Polyphen, SIFT and Mutation taster (pathogenicity prediction)	Variants were included if	
							NHLBI Exome Sequencing Project website release Version: v.0.0.9 (mutation frequency in ethnically matched controls)	- non-synonymous	
								- conservation score GERP >3	
								Confirmation *via* Sanger sequencing	
Krebs *et al.* (2013)	***SYNJ1* (Sac1- like inositol phosphatase domain of polyphosphoinositide phosphatase synaptojanin 1)**	Iranian family (healthy parents, who were first-degree relatives, as well as two affected, and three unaffected siblings)	Genome-wide SNP genotyping and homozygosity mapping was performed on a consanguineous PD family (HumanOmniExpress beadchips and HiScanSQ system, Illumina)	WES on two PD-affected siblings (HiSeq 2000, Illumina)	Burrows-Wheeler Aligner (BWA) tool (alignment of raw sequence reads to the human References genome - NCBI GRCh37)	GATK Unified Genotyper tool (SNP/SNV/InDel calling)	AnnTools (variant annotation)	Variants were excluded if	Homozygous c.773G > A
			Genome Studio program (genotyping quality assessment)		Genome Analysis Toolkit (GATK v1.5–16-g58245bf) (base-quality re-calibration and local realignment)		MutPred, SNPs&GO, Mutalyzer, HomoloGene (NCBI) and Clustalw2) (pathogenicity prediction)	- present in dbSNP137, 1,000 Genomes Project and Exome Variant Server of the National Heart, Lung, and Blood Institute (NHLBI) Exome Sequencing Project databases	- p. Arg258Gln (21q22.11)
			PLINK (Homozygous segment identification)					Variants were included if	
			Illumina genome viewer (homozygous segment visualizer					- located in exons or splice sites	
								Confirmation *via* Sanger sequencing	
Schulte *et al.* (2013)	***PLXNA4 (plexin A4)***	German Family	Genotyping of the top ten candidate variants (KORA-AGE cohort using	WES on 2 PD-affected second cousins. (Genome Analyzer IIx system (Illumina)	Burrows-Wheeler Aligner (BWA 0.5.8) (read alignment)	SAMtools (version 0.1.7) (SNV/InDel calling)	SIFT/PROVEAN, PolyPhen-2 and MutationTaster (pathogenicity prediction)	Variants were excluded if: observed in in-house exome database, dbSNP135, 1000-Genomes Project and NHLBI-ESP (EA only) databases with a minor allele frequency >1%	Heterozygous c.1970C > T
			MALDI-TOF masspectrometry on the SequenomH platform					Variants were included if	- p.Ser657Asn (7q32.3)
			Linkage analysis on 6 family members using oligonucleotide SNP arrays (500 K					- non-synonymous	
			Illumina)					- exonic/coding	
			MERLIN (Linkage analysis)					- missense, nonsense, stoploss, splice site or frameshift variants	
								Confirmation *via* Sanger sequencing	
Vilariño-Güell *et al.* (2014)	***DNAJC13* (receptor-mediated endocytosis 8/RME-8)**	Canadian (Dutch–German– Russian Mennonite) family	None	WES on three PD - affected members (Agilent SureSelect 38 Mb Human All Exon Kit, Illumina Genome Analyzer)	Bowtie 12.70 and Burrows-Wheeler Aligner (BWA 0.5.9) (read alignment to human References genome - NCBI Build 37.1)	SAMtools (variant calling)	SIFT (pathogenicity prediction)	Variants were excluded if	Homozygous c.2564A > G
					Genome Analysis Toolkit (GATk) (local realignment around insertions and deletions)			- Phred quality score <20	- p.Asn855Ser (3q22.1)
								- frequently observed in population databases (minor allele frequency >1%)	
								Confirmation *via* Sanger sequencing	
Funayama *et al.* (2015)	***CHCHD2* (coiled-coil-helix–coiled-coil-helix domain containing 2)**	Japanese family	Genome-wide linkage analysis on 8 affected and 5 unaffected individuals of the family (Genome-Wide Human SNP Array 6.0, Affymetrix)	WES on three patients & WGS on one patient (HiSeq 2000, Illumina)	Burrows-Wheeler Aligner (BWA-MEM version 0.5.9) (read alignment to References human genome - UCSC hg19)	SAMtools version 0.1.16 (SNV/InDel calling)	PolyPhen-2 & MutationTaster (pathogenicity prediction)	Variants were excluded if	Heterozygous 182C > T
			SNPHitLink & MERLIN (linkage analysis)					- present in the 1,000 Genomes, dbSNP138, the Human Genetic Variation database, and the National Heart, Lung, and Blood Institute (NHLBI) Exome Sequencing Project (ESP) database	- p.Thr61Ile (7p11.2)
								Variants were included if	
								- located in exons or splice sites	
								- heterozygous state	
								- non-synonymous or caused aberrant splicing	
								- located in regions with positive log of odds greater than 1	
								- not noted in unaffected Japanese controls	
								Confirmation by Sanger sequencing	
Sudhaman et al., (2016b)	***RIC3* (acetylcholine receptor chaperone)**	South Indian family	None	WES on a single index patient (HiSeq 2000, Illumina)	FastXToolkit (pre-alignment QC)	SAMTools and GATk (variant calling)	wANNOVAR (variant annotation)	Variants were excluded if	Homozygous c.169C > A
					Burrows-Wheeler Aligner (BWA) (read alignment)		KGGSeq (variant filtering)	- present in databases (dbSNP 135, 137 and 138, 1,000 genomes and National Heart, Lung, and Blood Institute (NHLBI) 6500 exomes and ExAC) with a MAF >0.01	- p.P57T (11p15.4)
					SAMTools (Post-alignment QC)			Variants were included if	
					BEDTools (assess target coverage and depth			- heterozygous	
								Confirmation *via* Sanger sequencing	
Sudhaman *et al.*, 2016a	***PODXL* (podocalyxn-like Gene)**	North Indian family	None	WES on two affected siblings (HiSeq 2000, Illumina)	FastXToolkit (pre-alignment QC)	SAMTools and GATk (variant calling)	wANNOVAR (variant annotation)	Variants were excluded if	Homozygous c.89_90 insGTCGCCCC
					Burrows-Wheeler Aligner (BWA) (read alignment)		KGGSeq (variant filtering)	- present in databases (dbSNP 135, 137 and 138, 1,000 genomes and National Heart, Lung, and Blood Institute (NHLBI) 6500 exomes and ExAC) with a MAF >0.01	- p.Gln32fs (7q32.3)
					SAMTools (Post-alignment QC)			Variants were included if	
					BEDTools (assess target coverage and depth			- homozygous (Autosomal recessive inheritance assumed)	
								- exonic variants	
								- shared between the two affected individuals	
								Confirmation *via* PCR-Sanger sequencing	
Deng *et al.* (2016)	***TMEM230* (Transmembrane Protein 230)**	Canadian-Mennonite (same family as DNAJC13)	None	WES on one unaffected individual and 4 distantly related affected cousins) (HiSeq2500, Illumina)	Genome Analysis Tool Kit (GATk v1.1) (read alignment to human References genome - Hg19)	Unified Genotyper from the Genome Analysis Tool Kit (SNV/INDEL calling and performing variant quality score (VQS) and Phred-likelihood scores)	ANNOVAR (variant annotation)	Variants were excluded if	Heterozygous c.422G > T
							PolyPhen2 (pathogenicity prediction)	- present in multiple databases including the dbSNP (v130), HapMap and 1,000 Genome databases with a MAF >0.01	- p.Arg141Leu (20p13-p12.3)
							SpliceView, NNsplice, and ESEfinder (splicing effect prediction)	- VQSLOD < −3	
								- alternate Phred-scaled likelihood scores <99	
								Variants were included if	
								- the average read per targeted base was >65X with the Phred quality score of ≥30	
								Confirmation *via* Sanger sequencing and co-segregation analysis	
Ruiz-Martinez *et al.* (2017)	***CSMD1* (CUB and Sushi multiple domains 1)**	Spanish Basque family	None	WES on index patient (HiSeq 2000, Illumina)	Burrows-Wheeler	GATK Unified Genotyper tool (SNP INDEL calling)	AnnTools kit (variant annotation)	Variants were excluded if	Heterozygous c.5885G > A
					Aligner Tool (BWA) (read alignment to the human References genome - NCBI		PICARD (Exome statistics)	- intragenic, intronic, and non-coding exonic	-p.Arg1962His
					GRCh37.p13)		MutPred, SNPs&Go, MutationTaster, and CADD (pathogenicity prediction)	- present in the dbSNP149 build, 1,000 Genomes	and c.8959G.A- p.Gly2987Arg)
					Genome Analysis		HomoloGene database (protein conservation across species)	Project phase 3, the Exome Variant Server of the National Heart, Lung, and Blood Institute (NHLBI) Exome Sequencing and the Exome Aggregation Consortium databases with a MAF >0.05	(8p23.2)
					Toolkit (GATK v1.5-16-g58245bf) (base-quality re-calibration and local realignment)		Human Gene Mutation database (HGMD) & NCBI	Variants were included if	
							ClinVar database (genotype-phenotype correlation)	- mapping quality (q30 or higher)	
								- depth of coverage (d10 or higher)	
Straniero *et al.* (2017)	***DNAJC12* (DnaJ Heat Shock Protein Family (Hsp40) Member C12)**	Canadian and Italian family	Positional cloning (Ion AmpliSeq™ Exome Kit and the Ion Proton™ System, Thermo Fisher Scientific)	WES on index patient (HiSeq 2000, Illumina)	Torrent Suite Software	Torrent Variant Caller (tvc 4.2-18) (variant calling)	ANNOVAR (variant annotation)	Confirmation, segregation analysis and screening *via* Sanger sequencing	Homozygous c.187A > T
									- p.K63* (10q21.3)
									and c.79–2A > G - p.V27Wfs*14 (10q21.3)
Quadri *et al.* (2018)	***LRP10* (Low-density lipoprotein receptor - related protein 10)**	Italian family	Genome-wide SNP array genotyping and linkage analysis in ten affected	WES on index PD patient (HiSeq 2000, Illumina)	Burrows-Wheeler Aligner (BWA-MEM version 0.5.9 (read alignment to human References genome - UCSC hg19)	Genome-Analysis-Tool-Kit (GATk) v3 (variant calling)	Cartagenia Bench Lab NGS v·5·0·1 (variant filtering)	Variants were excluded if	Homozygous
			Relatives (HumanCNV370 bead chip, Illumina)				SpliceSiteFinder-like, MaxEntScan, NNSPLICE, GeneSplicer, and Human Splicing Finder integrated in Alamut Visual version 4·2 (splicing effect prediction)	- present in dbSNP, Exome Variant Server NHLBI GO Exome Sequencing Project (ESP), 1000 Genomes, Genome of the Netherlands (GoNL), Exome Aggregation Consortium (ExAC) and the Genome aggregation database (GnomAD) databases with a MAF >0.01	- p.Gly603Arg (14q11.2)
			Copy number analysis (Nexus Copy Number, BioDiscovery)					Variants were included if	
			MERLIN (linkage analysis)					- heterozygous	
								- exonic	
								- non-synonymous	
								-within 5bp from a splice site	
								- predicted to be pathogenic with ≥5 in silico tools	
								Confirmation by Sanger sequencing	
Guo *et al.* (2018)	***NUS1* (Dehydrodolichyl Diphosphate synthase Subunit)**	Han Chinese family	None	WES on 39 EOPD patients (probands), their parents, and 20 unaffected siblings (HiSeq 2000, Illumina)	Burrows-Wheeler Aligner (BWA version 0.5.9-r16) (alignment to the human References genome - hg19)	HaplotypeCaller in GATk (SNV/InDel calling)	PolyPhen-2 (pathogenicity prediction)	Variants were excluded if	Heterozygous c.691+3dupA (6q22.1)
					Picard (marking of PCR duplicates)		DAPPLE (disease Association Protein-Protein Link Evaluator) (construction of protein-protein interaction networks)	- present in dbSNP137, the Han Chinese of 1,000 Genomes Project, or both of the two offspring in quads	
					GATk (InDel realignment recalibration of the base quality scores)		GEO2R (determine differential gene expression in protein networks)	- indels were in known structure variation regions	
							Gene Ontology (GO) (gene annotation)	Variants were included if	
							KEGG pathway database (functional enrichment)	- Phred quality scores >30	
							PLINK (single variant associations)	- there was only one type of alternative allele	
								- the read coverage of alternative alleles in the offspring was > than 4	
								- more than 30% and less than 5% of the covered reads were the alternative allele for the offspring and parents	
								- for the offspring: PL (0/0)≥30, PL (0/1) = 0, and PL (1/1)≥30 (PL: Phred-scaled likelihoods for a given genotype)	
								- for both parents PL (0/0) = 0, PL (0/1)≥30, and PL (1/1)≥30	
								- two adjacent SNVs were located at least 10 bp away	
								Confirmation of variants *via* Sanger sequencing	
(Lin et al., 2019)	***UQCRC1*** (***mitochondrial ubiquinolcytochrome c reductase core protein 1***)	Taiwanese Family	Custom-designed NGS Gene Panel (including 40 genes associated with parkinsonism) screening	WES on three affected individuals (Ion Torrent TM Next-Generation	Burrows-Wheeler Aligner (BWA-MEM) (alignment to the human References genome - GRCh37/hg19)	GATk (variant calling)	ANNOVAR (variant annotation)	Variants were excluded if	Heterozygous c.941A > C
				Sequencing Exon v2 kit and platform)	Picard (marking and removing duplicates)		CADD, PolyPhen-2 and SIFT (pathogenicity prediction)	- present dbSNP144, 1,000 Genomes Project, EXAC, gnomAD and the Taiwan Biobank with a MAF >0.01	- p.Tyr314Ser (3p21.31)
							Human Splicing Finder (splicing effect prediction)	Variants were included if	
								- exonic	
								Confirmation of co-segregation *via* Sanger Sequencing	
Sebate *et al.* (2021)	***NRXN2*** (***Neurexin-2***)	Afrikaner family (South Africa)	None	WES on three affected individuals (HiSeq 2000, Illumina)	Burrows-Wheeler Aligner (BWA-MEM) (alignment to the human References genome -GRCh37/hg19)	GATk (variant calling)	Annovar (variant annotation)	Variants were excluded if	Heterozygous p.G849D (C > T)
					SAMTools (mpileup) (read coverage statistics)		SIFT, PolyPhen-2, MutationTaster, CADD, GERP++ (pathogenicity prediction)	- present in the EXAC database, gnomAD, the 1,000 Genomes Project and dbSNP databases	(11q13.1)
							Allen Brain Atlas, Human Protein Atlas, KEGG database, PANTHER (pathway and expression analysis)	Variants were included if	
								- minimum Phred quality score >30	
								Confirmation *via* Sanger Sequencing	
Bentley *et al.* (2021)	***SIPA1L1* (Signal Induced Proliferation Associated 1 Like 1)**	Australian Families (family #002 and #433)	Probands were screened for known PD causes including SNVs and expansions of repetitive regions in ATXN2, ATXN3 and TBP, and copy number variations in SNCA and PARK2	#433 (*SIPA1L1*)	Torrent Suite (v4.0) was used for Ion Torrent data (alignment to the human References genome)	HaplotypeCaller from the GenomeAnalysis ToolKit (v3.5) for the MiSeq data (variant calling)	ANNOVAR (variant annotation)	Variants were excluded if	SIPA1L1-Heterozygous p.R236Q (14q24.2)
	***&***			WES on three PD-affected siblings (Ion AmpliSeq capture kit and sequenced using the Ion Torrent (Thermo Fisher Scientific, Waltham, MA, USA)	SamTools and bedtools2 (alignment to the human References genome)	Torrent Suite (v4.0) was used for Ion Torrent data (variant calling)		-seen in >30% of the MiSeq in-house datasets (2n = 48) or >0.5% of the AnnEx Annotated Exomes browser (2n = 5,902, https://annex.can.ubc.ca, accessed on 4 December 2020) for Ion Torrent data	KCNJ15 -Heterozygous p.R28C (21q22.13)
	***KCNJ15* (Potassium Inwardly Rectifying Channel Subfamily J**)			#002 (*KCNJ15*)		SamTools and bedtools2 (variant calling)		Variants were included if	
				WES on 2 PD-affected siblings and 2 PD-affected cousins and an unaffected cousin				- present in affected members of the family while taking into consideration incomplete penetrance	
				Illumina HiSeq, Illumina MiSeq and Ion Torrent)				- if were exonic or in a splicing region (RefSeq v61)	
								- missense allele	
								- minor allele frequency of <0.01 in the gnomAD database	
								Confirmation *via* Sanger Sequencing	

In 2011, Vilariño-Güell and others published their WES findings on two first degree cousins from an AD PD-affected Swiss family, announcing the discovery of the p.Asp620Asn mutation in *VPS35* (Vilariño-Güell et al., 2011). In a back-to-back publication, that same mutation in *VPS35* was also identified in an Austrian family (Zimprich et al., 2011). Their study made use of haplotyping and linkage analysis in conjunction with WES, allowing for the simultaneous identification of linkage regions and the subsequent filtering of variants based on their distance to the linkage regions. Thus, postulating a time-and cost-effective approach to exome sequencing for AD-PD (Bras and Singleton, 2011; Gialluisi et al., 2020). Furthermore, the same mutation was found in six unrelated PD individuals of varying ethnicity and observed in a sporadic PD case (Zimprich et al., 2011). With these findings in several independent PD families, *VPS35* is now considered a significant gene associated with AD-PD, though with still unresolved pathology. The successes observed in these two early studies sparked hope for the discovery of rare monogenic causal factors using NGS in PD families and subsequently, several similar studies ensued.

In 2012, the discovery of *DNAJC6,* linked to AR-juvenile parkinsonism in a consanguineous Palestinian family, was published (Edvardson et al., 2012). They performed SNP genotyping and homozygosity mapping (HM) analysis in conjunction with WES (Edvardson et al., 2012; Vahidnezhad et al., 2018). This approach potentially facilitates more rapid detection of a disease gene after WES (Kim et al., 2013). HM analysis allows for the identification of large, shared regions of homozygosity (where variants associated with AR disease genes are likely to be located) between affected family members (Wakeling et al., 2019). Therefore, HM could be beneficial for the identification of pathogenic mutations in AR-PD (Bras and Singleton, 2011). The following year, the same approach on a consanguineous Iranian family affected with early-onset PD (EO-PD) led to the discovery of a homozygous mutation in *SYNJ1* (Krebs et al., 2013). Also in 2013, the finding of a heterozygous p.Ser657Asn mutation in *PLXNA4* within a large German family, was published (Schulte et al., 2013).

Vilariño-Güell and others published their findings on identification of the p.Asn855Ser mutation in *DNAJC13* in 2014 (Vilariño-Güell et al., 2014). WES was conducted on a large PD-affected Canadian-Mennonite family of Dutch German-Russian ancestry. The same mutation and disease-associated haplotype was found in two other families of Mennonite ancestry in the greater Canadian region (Vilariño-Güell et al., 2014). Remarkably, another group, studying the original Canadian-Mennonite family, published their findings in 2016, on a different genetic causal variant, p.Arg141Leu in *TMEM230* (Deng et al., 2016). This difference in disease gene nominations in the same family may be due to differences in methodological approach, including the clinical phenotype used, genotyping approach and pathogenicity prediction scoring of mutations (Farrer, 2019). This highlights the importance of accurate clinical information, particularly in a disease like PD, where the phenotype may overlap with related neurological disorders.

Notably, in the discovery of *CHCHD2* in 2015 in AD-PD, Funayama *et al.*, performed both WES and WGS (Funayama et al., 2015). The authors state that WGS was done on one affected family member to correct for the regions that were inadequately covered during exome capture (Funayama et al., 2015). The use of WGS in combination with WES (particularly in the individual who has the variant of interest) is considered highly beneficial due to its increased coverage and enables screening for CNVs/SNVs in the regions of interest. However, WES continues to be the sequencing method of choice (and was the sole NGS approach used in 16/17 of the studies in Table 1), which could largely be attributed to the significant disparity in cost.

In 2016, Sudhaman and others nominated *RIC3* (Sudhaman et al., 2016a) and *PODXL* (Sudhaman et al., 2016b) in South Indian and North Indian families, respectively. For *RIC3*, microsatellite markers were used, prior to WES, to rule out linkage to known AD-PD genes including *SNCA*, *LRRK2* and *VPS35* (Sudhaman et al., 2016a). A similar approach was used to discover *PODXL*. In 2017, a study using WES on a Spanish Basque family led to the discovery of *CSMD1* as a potential disease-causing gene (Ruiz-Martínez et al., 2017). That same year, another study reported a homozygous loss-of-function mutation in *DNAJC12,* using a positional cloning approach in combination with WES (Straniero et al., 2017).

In 2018, two more novel PD genes were reported. In one study, SNP genotyping, linkage analysis, CNV analysis and WES was used in an Italian family to identify the Gly603Arg mutation in *LRP10* (Quadri et al., 2018). In PD, *de novo* mutations may potentially account for several sporadic, EO-PD cases. In the second study, WES and subsequent analysis was performed on trios of Han Chinese ancestry with EO-PD and identified potential pathogenic *de novo* mutations in *NUS1* (Guo et al., 2018). *De novo* mutations are typically rare, deleterious, and difficult to detect with traditional genotyping methods but were effectively identified using only WES in this study (Wang et al., 2019).

In 2019, the identification of *UQCRC1* (a nuclear-encoded gene associated with mitochondrial metabolism) implicated in a Taiwanese PD family with parkinsonism and polyneuropathy, was published (Chen and Lin, 2020; Courtin et al., 2021). This study was the only one to make use of a comprehensive NGS gene panel to pre-screen ∼40 PD-associated genes (including *SYNJ1, DNAJC13, DNAJC6, CHCHD2*, *VPS35*) before performing WES*.* A study published in 2021 described the discovery of a novel PD gene (*NRXN2*) in a family from South Africa (Sebate et al., 2021). They analyzed WES data from 3 affected individuals from an Afrikaner family, an ethnic group consisting of Dutch, German and French ancestry that are native to South Africa. Most recently, a study examining six families from Australia used WES to narrow down two novel potential disease-causing genes in two families - *SIPA1L1* and *KCNJ15* (Bentley et al., 2021)*.*

It should be noted that true monogenic PD is rare and establishing a familial PD candidate gene as pathogenic can have a degree of uncertainty due to the following factors: isolated findings in familial studies, presence of disease variants in healthy controls, erroneous gene-disease associations or possession of complex phenotypes that may skew towards other, diverse parkinsonisms (Day and Mullin., 2021). Of the candidate genes outlined in this article, VPS35, otherwise referred to as *PARK 17*, is firmly associated with classical PD. However, *DNAJC6* (*PARK 19*), *DNAJC13* (*PARK 21*), *SYNJ1* (*PARK 20*), *VPS13C* (*PARK 23*), and *CHCHD2* (*PARK 22*) are also considered pathogenic and viewed as rare genetic contributors to PD disease (Olgiati et al., 2016; Puschmann, 2017; Schormair et al., 2018; Correia Guedes et al., 2020; Day and Mullin., 2021; Li B et al., 2021). The remaining candidate genes require further study before being categorized as definite PD genes. “Proof of pathogenicity” of novel disease genes require that multiple mutations in the same gene co-segregate with disease in independent families, are absent in large collections of healthy controls or found to be significantly associated with sporadic PD cases (MaCarthur et al., 2014; Farrer, 2019). These criteria seem to necessitate a move away from small family studies and into population-based NGS studies for rare variant discovery - once again relying on large cohorts of individuals. This is also supported by the reasoning that many PD loci may be population-specific and therefore difficult to identify in small studies The (International Parkinson Disease Genomics Consortium, 2020). However, confirmation of these putative mutations through functional studies or by utilizing model organisms remains a challenge due to the novelty and the large number of variants being identified through NGS.

Consequently, it is clear that there is still a need for NGS studies on PD-affected families for its ability to nominate potentially pathogenic novel genes, even if not seen in other individuals, as this may provide mechanistic insight into PD pathobiology. As seen with the discovery of NUS1, where knockout RNAi experiments on *Drosophila* revealed PD phenotypes, lab-based functional analysis of candidate genes is useful to uncovering disease pathogenesis (Guo et al., 2018). However, many studies omit lab-based functional analysis due to the uncertainty as to whether the gene is disease-causing (Rodenburg, 2018). Alternatively, candidate genes can be further associated with a disease of interest through phenotypic associations, determining gene or protein interaction networks or establishing functional similarity with known PD genes using computational methods (Chen et al., 2021). Increasingly, a number of machine learning methods that incorporate information from known databases that provide functional annotations (e.g. Gene Ontology), tissue expression data (e.g., Human Protein Atlas) and metabolic/signaling pathways (e.g., Kyoto Encyclopaedia of Genes and Genomes) in order to determine protein or gene interactions between putative and established disease genes (Piro and Di Cunto, 2012). According to a recent study outlining a comprehensive PD gene database (*GENE4PD*), a functional correlation network was simulated between “high confidence” and “suggestive” PD-associated genes in PD pathways resulting in significant associations, including those seen with *RIC3* and *CHCHD2*, with the latter significantly linked to *SNCA, PINK1, LRRK2, PARK7*, and *VPS35* - a likely potential for expanding our knowledge on PD pathway architecture and future annotations (Li B et al., 2021). Furthermore, it is difficult to characterize a gene as being only PD-associated due to the inter-lapping of disease pathways across various parkinsonism disorders (Erratum, 2019; Li W et al., 2021).

### Analysis of Bioinformatic Pipelines Used in PD Genomic Studies

**FIGURE 1:**
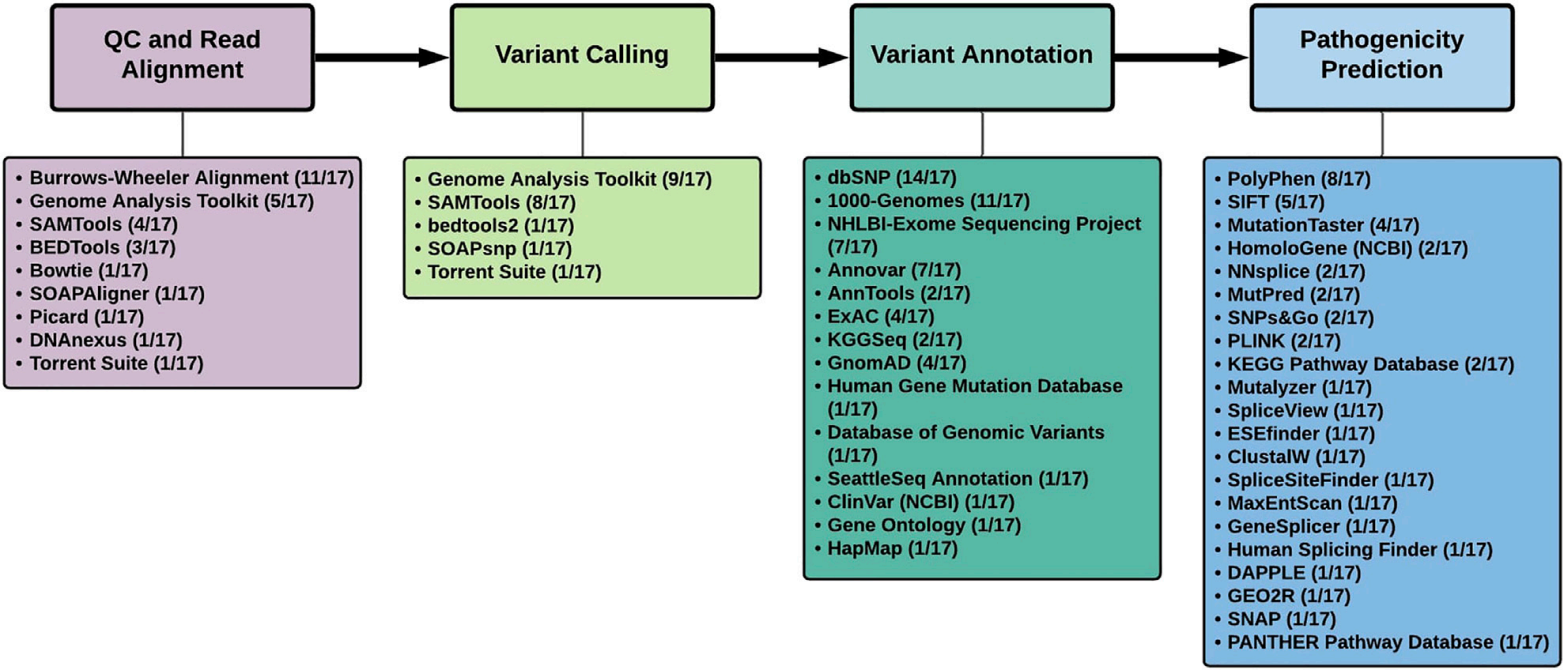
Summary of tools used to analyze next-generation sequencing data in the 17 studies that identified novel Parkinson’s disease genes.

Analysis of the tools used in the 17 studies, revealed several similarities and differences (Table 1; Figure 1).

Burrows-Wheeler Aligner (http://bio-bwa.sourceforge.net/), specifically the BWA-MEM algorithm, was the software of choice (11/17 studies) for the alignment of the NGS reads to the human reference genome [Figure 1]. The studies reviewed here made use of both the hg18/GRCh36 and hg19/GRCh37 reference genomes. According to one study, SNV detection in WGS data resulted in enhanced genome coverage and a higher number of SNV calls when using GRCh38, as opposed to GRCh37, thereby necessitating the use of the latest reference genome available for NGS analysis (Pan et al., 2019). They conclude that the selection of the aligner in NGS is not as important as the reference genome selection (Pan et al., 2019). The UnifiedGenotyper was used for variant calling in 7 of the 9 studies using the Genome Analysis Toolkit (GATk). This was until the more recent studies, including *NUS1, NRXN2* and *KCNJ15,* made use of GATk’s HaplotypeCaller for variant calling (Guo et al., 2018). The HaplotypeCaller is now considered best practice for variant calling through GATk’s Best Practices Workflows (https://gatk.broadinstitute.org) as it allows for SNP/inDEL detection *via de novo* haplotype assembly (Odumpatta & Mohanapriya. 2020). However, a combination of variant callers may be the most efficient method to prioritize variants (Kumaran et al., 2019; Zhao et al., 2020).

Annovar (https://annovar.openbioinformatics.org/) and AnnTools (http://an-ntools.sourceforge.net/) were the annotation tools used most frequently in 7/17 and 2/17 studies, respectively (Figure 1). These tools are capable of annotating variants using either gene-based, region-based or filtering-based approaches. A typical exome will produce ∼20,000 variants with ∼10% of these being novel (Belkadi et al., 2015). Thus, the variant filtering tools and exclusion/inclusion criteria must be sufficiently sensitive to identify the most likely causal factors from the ‘background noise’ (Kalinderi et al., 2016). In these PD studies, variants were searched against specific databases to determine allele frequencies. As seen in Figure 1, the three most frequently used databases are dbSNP (14/17), the 1000-Genomes-Project (11/17) and the NHLBI - Exome Sequencing Project (7/17), which are currently still considered the most widely used databases for NGS analysis. It was noted that GnomAD, the largest open-source population database, was only mentioned in 4/17 studies and highlights the need to prioritize the use of the larger databases (including the newly released UK BioBank database (https://www.ukbiobank.ac.uk/) as it may affect minor allele frequency (MAF) scores used in downstream variant filtering. Several criteria exist to prioritize possible disease-causing variants (Karczewski et al., 2020). Variants are excluded if they are synonymous as they are typically considered to be evolutionary neutral and are likely to have no functional impact on the protein. Variants are also excluded if found to appear in public databases with a MAF >0.01 indicating that the alternate allele is present in more than 1% of the population and is therefore a polymorphism. However, for inclusion, variants must possess PhRED scores >30 (indicating a base call accuracy of 99.9%), be exonic (at present, variants of interest are localized to protein-coding regions as disease-causing variants are likely to impact protein function), have either heterozygous or homozygous genotypes specific to the Mendelian inheritance pattern observed in the family, and also be validated through Sanger sequencing (Vilariño-Güell et al., 2011).

Notably, several caveats need to be considered in the case of PD. Homozygous variants may be disease-causing but may commonly appear in databases such as dbSNP and the 1,000 Genomes Project in heterozygous form, and therefore may be filtered out before variant prioritization (Bras & Singleton., 2011). Furthermore, there are instances in which not all PD affected family members carry the same pathogenic mutation and present as phenocopies (whereby two affected PD individuals with matching phenotypes in a family have different genotypes possibly due to an environmental risk factor). This phenomenon can easily be confused with intrafamilial heterogeneity (where one affected individual has a different mutation to the family mutation but where this difference may be due to *de novo* mutations, epigenetic changes, or pleiotropy or, in another instance, where multiple rare variants contribute to individual disease risk as seen in oligogenic inheritance (Klein et al., 2011; Farlow et al., 2016; Bentley et al., 2021). True phenocopies in a family may also lead to incorrect conclusions regarding the inheritance pattern within the family (Klein et al., 2011). These confounding factors are relevant in PD, thus requiring adaptation of inclusion criteria in bioinformatic tools going forward.

Popular tools used in these studies to predict the pathogenicity of variants included SIFT (https://sift.bii.a-star.edu.sg/) (5/17) and PolyPhen-2 (http://genetics.bwh.har-vard.edu/pph-2/) (8/15) (Flanagan et al., 2010). SIFT determines the effect of amino acid substitution on the protein function whereas PolyPhen-2 predicts the structural and functional impact non-synonymous SNPs have on the protein based on phylogenetic analysis (Odumpatta and Mohanapriya. 2020). Furthermore, many of the other pathogenicity prediction tools in Figure 1 were aimed at identifying variants with splice site effects. Subsequent performance assessment of pathogenicity assessment tools identified other options that outperform PolyPhen-2 and SIFT (Niroula and Vihinen, 2019). Recently, it has been noted that deep neural network models, in conjunction with general pathogenicity predictors such as CADD, are capable of improved variant prioritization as opposed to using the tool alone (Rentzsch et al., 2021). This may open the door to novel machine learning approaches, tailored to the disease of interest, in identifying or confirming disease-causing genes. Many of these newer tools, including RENOVO (Favalli et al., 2021) and DeepPVP (Boudellioua et al., 2019), typically make use of phenotypes to identify gene-disease associations by employing the use of publically available databases including ClinVar.

Also, there is a push to validate the functionality of these novel genes with wet-laboratory-based methods. However, the development of bioinformatic tools to aid the functional analysis of candidate variants may be useful in the interim. VS-CNV (Fortier et al., 2018), dudeML (Hill and Unckless, 2019), CNV-MEANN (Huang et al., 2021) are examples of newer computational software developed to detect and call CNVs in NGS data (including both exome and gene panel data) with CNVnator (Abyzov et al., 2011), Control-FREEC (Boeva et al., 2012) and LUMPY (Layer et al., 2014)) still widely used to replace standard multiplex ligation-dependent probe amplification (MLPA), fluorescence *in situ* hybridization (FISH) or microarray CNV detection (Zhang et al., 2019). In the discovery of *NRXN2*, computational protein modelling was performed using the Swissmodel webserver to simulate the potentially disruptive effect of the mutation on protein structure (Sebate et al., 2021).

### NGS Approaches to Study PD Genetics in Sub-saharan Africa

As observed for LRRK2 p.G2019S, some PD-causing mutations may be population-specific (Correia Guedes et al., 2010). Therefore, given the significant differences in ancestral origins, it is likely that the genetic etiology of sub-Saharan populations may be different to that of European and Asian populations (Bope et al., 2019). Mutation screening of Sub-Saharan African individuals with PD has revealed a low frequency in the known PD-causing genes, thus fueling this hypothesis (Williams et al., 2018; Dekker et al., 2020). Additionally, a recent study, using commercial MLPA kits to detect CNVs in individuals with PD from South Africa and Nigeria, observed false-positive deletions due to the presence of SNPs, highlighting the need for data from diverse populations when designing genomic assays for detecting PD mutations (Müller-Nedebock et al., 2021).

The current human reference genome build (GRCh38) is derived from a small sample size, with ∼70% of the build derived from a single donor of European ancestry, thereby lacking genetic diversity and therefore inadequate in the context of genetic research in Africa (Wong et al., 2020). Attempts to bridge this fundamental gap in African genomics are currently underway. An example is the South African Human Genome Project initiative to develop a local reference genome based on 24 African ancestry individuals (https://sahgp.sanbi.ac.za/). Another initiative is the H3Africa Consortium which aims to develop a pan-African bioinformatics network (H3ABionet) and infrastructure to enhance African genomics research on the continent (Mulder et al., 2017). Additionally, South African researchers have developed a secondary data analysis pipeline to overcome the lack of African allele frequency data in population databases (Schoonen et al., 2019). Their software incorporates Ensembls Variant Effect Predictor (https://www.ensembl.org/info/docs/tools/vep) to annotate variants and GEnome MINIng (GEMINI v0.20) (https://gemini.readthedocs.io/) to effectively filter variants according to African allele frequencies, resulting in higher quality output (Schoonen et al., 2019). Furthermore, international efforts in PD are underway to bring underrepresented populations to the fore, through standardized NGS data storage and analysis, as seen with the Global Parkinson’s Genetics Program (Global Parkinson’s Genetics Program., 2021) that aims to sequence and analyze PD-affected, at-risk and control individuals from diverse populations to bridge the gap in the ‘missing heritability’ witnessed in PD.

Recently, the exponential increase in large genomic datasets has necessitated the use of cloud-based systems for the ease of storage, analysis and data-sharing (Navale and Bourne, 2018). However, cloud-based systems can be expensive and require careful consideration of the data use policies to adhere to security in the cloud. Another glaring issue in computational biology is inconsistencies regarding the reproducibility of genomic data analysis and reuseability of open-source analytic software (Russell et al., 2018). A review examining the state of Github repositories of popular bioinformatic tools found that nearly half (46%) of all public repositories had no opensource license and nearly 12% had no version control (Russell et al., 2018). They suggested that software need to be vetted for consistent maintenance by a developer team. Thus, it is important to check the credibility of analysis software before use in a research or clinical setting, and a need for journals to insist on providing datasets and code to reproduce analyses.

### Future Directions and Conclusions

The initial studies that discovered *VPS35*, created excitement about the subsequent elucidation of the genetic etiology of PD. However, that initial hope has not been realized with most of the genes identified through NGS, only being found in a single family. This may be due to the complexity of PD etiology, with either, each family having its own rare genetic cause, or that the more common genetic causes underlying PD have not yet been identified. This leads us to question the future direction of NGS approaches in PD.

Third-Generation Sequencing or long-read sequencing are newly-developed approaches that aim to overcome the limitations of existing NGS methods. They produce long-reads that are far more expansive, reducing the complexity of detecting read overlaps—thus increasing the quality of the sequencing data and improving CNV detection (Giani et al., 2019). Approximately 15% of the genome is assumed to be inaccessible due to atypical GC content and repeat elements including trinucleotide repeat expansions which are disease-causing in several neurological disorders, including PD (Keogh and Chinnery., 2013). These mutable regions may harbor pathogenic mutations, particularly compound heterozygous mutations that may only be discovered with long-read sequencing (Mantere et al., 2019). Another limitation of short-read lengths produced by traditional NGS, is potential misalignment of *GBA* (a common genetic risk factor for PD) to its pseudogene which is ∼96% identical*,* resulting in false-positive mutations (Bras and Singleton., 2011). Furthermore, a study that explored the use of targeted-capture long-read sequencing of *SNCA* transcripts, detected previously-undiscovered isoforms capable of translating novel proteins (Tseng et al., 2019). Therefore, in the near future, long-read sequencing may be viewed as the more favorable sequencing alternative for disorders such as PD.

In conclusion, determining the complex genetic architecture underlying PD, particularly in underrepresented populations, is critical to provide insight into PD molecular mechanisms, detection of PD biomarkers, and elucidation of novel drug targets. Thus, this knowledge will change the course of future clinical diagnoses and therapeutic modalities for this currently incurable disorder. The aim of this article was to explore the use of NGS approaches to identify novel candidate genes in familial PD to consider not only their current relevance in research, but also their future potential in unraveling PD genetics. From our analysis, we recommend the use of third-generation sequencing technologies to identify complex genomic rearrangements and new sequence variation, in combination with current sequencing techniques, to propel future PD genetics research. Furthermore, we recommend that NGS researchers optimize and adjust their sequencing and analytic workflows according to the genetic background of their study participants with PD, and the constant evolution of bioinformatic tools. NGS approaches have revolutionized novel disease gene discovery, however, best practice guidelines need to be developed; taking into account diverse populations and ancestral origins, since it is apparent that a “one-size-fits-all” approach will have significant limitations.

## Data Availability

The original contributions presented in the study are included in the article/Supplementary Material, further inquiries can be directed to the corresponding author.
